# Revisiting the epidemiology of pertussis in Canada, 1924–2015: a literature review, evidence synthesis, and modeling study

**DOI:** 10.1186/s12889-020-09854-4

**Published:** 2020-11-20

**Authors:** Edward Thommes, Jianhong Wu, Yanyu Xiao, Antigona Tomovici, Jason Lee, Ayman Chit

**Affiliations:** 1 Sanofi Pasteur, Toronto, ON Canada; 2 University of Guelph, Guelph, Ontario Canada; 3 York University, Toronto, Ontario Canada; 4 University of Cincinnati, Cincinnati, OH USA; 5 Leslie Dan Faculty of Pharmacy, University of Toronto, Toronto, Ontario Canada

**Keywords:** Pertussis, Pertussis vaccines, Epidemiology, Modeling, Canada, National surveillance

## Abstract

**Background:**

Disease surveillance is central to the public health understanding of pertussis epidemiology. In Canada, public reporting practices have significantly changed over time, creating challenges in accurately characterizing pertussis epidemiology. Debate has emerged over whether pertussis resurged after the introduction of adsorbed pertussis vaccines (1981–1985), and if the incidence fell to its pre-1985 after the introduction of acellular pertussis vaccines (1997–1998). Here, we aim to assemble a unified picture of pertussis disease incidence in Canada.

**Methods:**

Using publicly available pertussis surveillance reports, we collected, analyzed and presented Canadian pertussis data for the period (1924–2015), encompassing the pre-vaccine era, introduction of vaccine, changes to vaccine technology, and the introduction of booster doses. Information on age began to be reported since 1952, but age reporting practices (full, partial or no ages) have evolved over time, and varied across provinces/territories. For those cases reported without age each year, we impute an age distribution by assuming it follows that of the age-reported cases.

**Results:**

Below the age of 20 years, the adjusted age-specific incidence from 1969 to 1988 is substantially higher than existing estimates. In children < 1 year, the incidence in some years was comparable to that during the 1988–1999 resurgence.

**Conclusions:**

The results presented here suggest that the surge in the average yearly incidence of pertussis that began in 1988 was weaker than previously inferred, and in contrary to the past findings, below age 5, the average yearly incidence of pertussis from 1999 to 2015 (when the incidence dropped again) has been lower than it was from 1969 to 1988.

## Background

*Bordetella Pertussis* is a highly contagious bacterium that transmits between all age groups [1, 2]. On average, a person infected with this bacterium can infect up to 10–15 other persons [3]. Infection may lead to serious respiratory complications, permanent neurological sequelae, or even death [3, 4]. The disease is most severe among infected infants too young to receive vaccination. Acoording to [3], about half of babies younger than 1 year old who get pertussis need care in the hospital, and among those babies treated in the hospital with pertussis, 23% get pneumonia, 1.1% will have convulsions, 61% will have apnea, 0.3% will have encephalopathy, and 1% will die. Protection acquired against infection and disease – whether from vaccination or from previous infection – is not life-long and wanes over time. This means that groups of individuals susceptible to infection can build up overtime creating the conditions to facilitate community outbreaks, especially given the highly contagious nature of the bacterium [4]. The public health response to such outbreak’s hinges on the availability of quality epidemiological surveillance data.

The nature of pertussis disease surveillance in Canada has evolved over time. The first records trace back to 1880 and were constrained to documenting pertussis related deaths. Subsequently records of severe disease mostly leading to hospital admission emerge, and recently records capture milder forms of laboratory confirmed disease. The laboratory testing technologies have also evolved over time, with culture methods used in the early years and now polymerase chain reaction (PCR) technology. Regardless of time and laboratory technology, public health surveillance has predominantly been passive with the majority of reported cases being severe enough that they present to healthcare.

Pertussis became a nationally notifiable disease across Canada in 1924 [5]. However, some provinces collected pertussis-related data long before then. For instance, as early as 1880 Ontario recorded pertussis deaths and in 1905 added records on pertussis related hospital admission [6, 7]. In contrast, the three territories did not report data publicly until the late 1960’s and Nunavut only started in the late 1980’s. The federal government started systematically collating provincial data sets and publicly reporting on the national number of pertussis related cases and deaths stratified by age and sex in 1952. Table 1 in the next section gives a summary of published reports/studies describing the trends in incidence reports and their interpretations. It is clear that the national data have been subject to updates and corrections due to issues such as late and missed records. Despite this, it remains challenging to review the full history of pertussis epidemiology in Canada due to the evolution of surveillance and reporting methodology over time.

**Table 1:** Summary of reports/studies describing the trends in incidence reports and their interpretations. (diff = difference observed, no-diff = no difference observed, age-incidence = age-stratified incidence)

Publication Source, year	Years of data	Place(s) of data	Type of data	Interventions discussed and conclusions
Ross [6], 1932	1880–1929	Ontario	Mortality	inter-disease (diff), male/female (no-diff), urban/rural (no-diff), age-group (diff).
Museum of Health Care [7]	1880–1934 1905–1934	Ontario	Mortality Morbidity	The incidence data was not used in practice.
Varughese et al. [8], 1979	1924–1978 1960–1978 1969–1976	Canada	Total incidence Age-incidence Hospitalization	Incidence declined after vaccine introduction in 1943, as expected.
Varughese et al. [9], 1985	1924–1984 1960–1984 1980–1981	Canada	Total incidence Age-incidence Hospitalization	Hospitalization rates and incidence rates were almost equal, meaning that incidence reports are incomplete.
Halperin et al. [10], 1989	1985–1987	Nova Scotia	Age-incidence	The use of enhanced surveillance showed patterns of incidence similar to pre-vaccine. Whole-cell vaccine was not very effective.
Skowronski et al. [11], 2002	1981–2000	British Columbia	Age-incidence	Poor whole-cell vaccine created a cohort effect. Switch to more effective acellular changed the epidemiology. Introduction of PCR resulted in increased incidence report.
Ntezayabo et al. [12], 2003	1983–1998	Quebec	Age-incidence	Cohort effect, caused by poor whole-cell vaccine, was observed.
Galanis et al. [13], 2006	1924–2002 1988–2002	Canada	Total incidence Age-incidence	Switch to acellular vaccine reversed observed resurgence. Cohort effect predicted caused by adolescent booster introduction. Adult booster would protect against transmission from adults to their contacts.
Vickers et al. [14], 2006	1995–2005	Saskatchewan	age-incidence	Whole cell or combined whole-cell/acellular worked better than pure acellular.
Bettinger et al. [15], 2007	1991–2004	Canada	Hospitalization	Switch from adsorbed whole-cell to acellular improved protection of small children but did not change incidence of infants. 1-dose adolescent or adult booster suggested to reinforce indirect protection to infants.
Greenberg et al. [16], 2009	1988–2004 1991–2006	Canada	age-incidence hospitalization	Both combined DTap-Hib and adolescent/adult Tdap offered effective protection against pertussis.
Fisman et al. [17], 2011	1993–2007	Greater Toronto Area	Culture and PCR test records	Proposed a feedback model where increasing test positivity led to increased test submissions. Seasonality may be due to cough symptom interference/misdiagnosis.
Smith et al. [18], 2014	1924–2012 1980–2012 1991–2012 1991–2011	Canada	total incidence age-incidence hospitalization hospitalization	The incidence trends followed expectation from vaccinations. 2012 rise was unexpected. Variations in incidence varied by province and territory. Enhanced future monitoring was suggested.
Chambers et al. [19], 2014	1993–2013	British Columbia	age-incidence	Ratio of positive tests to overall test did not change much even in outbreaks, supposedly because of improved reporting. Improved future reporting was suggested.
Government of New Brunswick Report [20], 2014	2012 2006–2013	New Brunswick	age-incidence region-incidence	Age groups 10-14y had the highest incidence due to waning. Booster catch-up campaigns and adolescent (any age)/adult booster for those in contacts with infants implemented/recommended.
Deeks et al. [21], 2014	2011–2013	Ontario	age-incidence for religious community/general population	Age profile of pertussis in religious under-immunized community resembled prevaccine era. Many cases in immunized 10-14y was considered a sign of waning of vaccine protection.
Liu et al. [22], 2017	2004–2015	Alberta	age-incidence zone-incidence	Outbreaks detected based on comparison with baseline incidence in 2008 and 2012. Majority of cases had not received adequate vaccination.

The case definition for a confirmed case has evolved as well. The national pertussis case definition for a confirmed case has changed based on the following timeline since 1991. From 1991 to 2000, the case definition for a confirmed case consisted of isolation of *B. pertussis* and presence of a clinically compatible symptom [23, 24]. In 2000, the case definition for a confirmed case was modified so that laboratory diagnosis was expanded to include detection of DNA with PCR testing [19, 24]. From 2000 to 2008, the national case definition required laboratory confirmation (via culture from an appropriate clinical specimen or detection of DNA with PCR) or an epidemiological link to a laboratory confirmed case and one or more of three clinical symptoms [23, 25]. In 2008, the case definition was modified to require cases in which *B. pertussis* DNA was detected to also have one or more of four clinically compatible symptoms [23, 26].

Vaccination programs have lowered the level of reported disease in Canada over time. Primary reasons amongst these was the 1943 introduction of mass vaccination campaigns of infants and small children using a whole cell, inactivated pertussis vaccine. This vaccine was switched to an adsorbed whole cell vaccine during the period 1981–1985, which was further replaced by the less reactogenic, acellular formulation in 1997. Vaccination boosters in adolescents were introduced between 1999 and 2004. Vaccine coverage changes over the year, according to the 2017 survey [27], 76% of two-year old children had received all recommended doses (four) of diphtheria, tetanus and pertussis vaccine, still far below the national goal of 95% for all recommended childhood vaccines. This coverage also varies by age milestones.

Surveillance data are key to managing and assessing the success of these public health interventions, and to evaluate the impact of immunization programs and changing vaccine products. However, researchers have yet to reach a full consensus in interpreting temporal trends in Canadian pertussis epidemiology. Assessing the incidence data with age information is a key step towards better modelling and analyzing the temporal trends in order to reach a full consensus. Here, we assemble and present historical incidence data collected in Canada for the period from 1924 to 2015, while attempting to impute missing age information. It is our hope that the data and the discussion in this paper will help to further the understanding of pertussis epidemiology in Canada.

## Methods

Canadian pertussis surveillance data has informed a substantial body of research, stretching as far back as the 1930s. We conducted a targeted literature review of government reports and published studies involving Canadian pertussis surveillance and/or the interpretation thereof. Results of the review are summarized in Table 1; the studies comprise a mix of passive and active surveillance. For each study, the table lists the location and timespan of the incidence data, the relevant intervention(s) considered, and interpretations. See Appendix A3 for a more detailed summary of each report/article. The majority of studies interpreting surveillance data do so in the context of assessing vaccine performance. One recurring theme is a dramatic resurgence of pertussis beginning in 1989. In almost all cases, this is interpreted as having been due to inferior protection provided by the adsorbed whole-cell pertussis vaccine that served as the intermediate replacement of whole-cell vaccine in Canada. Assessment of the performance of the subsequently introduced acellular vaccine is less clear-cut; it is variously concluded that it has performed worse than the original whole-cell vaccine, or better only in older children. The possibility is also raised that observed changes in the disease burden stem in part from changes in testing practices. Here, we focus on another factor influencing pertussis surveillance, namely provincial and temporal variability in how ages of cases have been reported.

### Reported incidence of pertussis in Canada by age group and year, 1952 to 2015

To fully cover the period from 1952 to 2015, we needed to extract incidence data from several sources, each covering a different part of this timespan: *National Notifiable Diseases* yearly published reports (1991–2015) [28], Smith et al. (1980–1990) [18], Varughese (1979) [8], and *Annual Report of Notifiable Diseases* yearly published reports (1952–1978) [29–31]. More details on the sources are given in Supplement 1.

We found that up until 1959, age stratified surveillance reports presented data for children below 1 year of age, children 1–5 years, 5–14 years, 15–19 years, and adults 20 years of age or older. Between 1959 and 1971 data on children 5–14 years of age were further stratified into two separate groups 5–9, and 10–14 years of age. Adults were stratified into 20–39, 40–59, and 60 years of age and older. Adults were further stratified in 1972 to 20–24, 25–29, 30–39, 40–59, and ≥ 60 years of age and older. The reasons underlying the initial selection and subsequent revisions were not given explicitly. However, the change may reflect public health concerns in the post-vaccine era.

### Calculation of rates

With these data, one can directly calculate only the age-stratified yearly incidence rates for those cases reported with age information, as has been done in the past. However, since our aim was to estimate age-stratified rates including all reported cases, imputation of missing ages was required. Below, we describe our methodology for doing this.

To begin with, we calculated the overall rates of yearly incidence across all ages, using the reported yearly incidence totals and historical population data available from Statistics Canada [32]:

1 $$ R(i)=\frac{C(i)}{P(i)},i=1924,\cdots, 2015 $$

where *R*(*i*) is the calculated incidence rate, *C*(*i*) is the number of reported cases, and *P*(*i*) is the total population, each in year *i*.

Before 1952, pertussis reporting in Canada did not include age information, so that only the overall rate *R*(*i*) could be calculated. Reporting of ages began in 1952, but with reporting practices changing over time in each province (see Table S3), each year there continued to be a proportion of cases *C*^*U*^(*i*) without age information. These have typically been reported in the form of an “age-unknown rate”,

$$ {R}^U(i)=\frac{C^U(i)}{P(i)}. $$

**Fig. 1:**
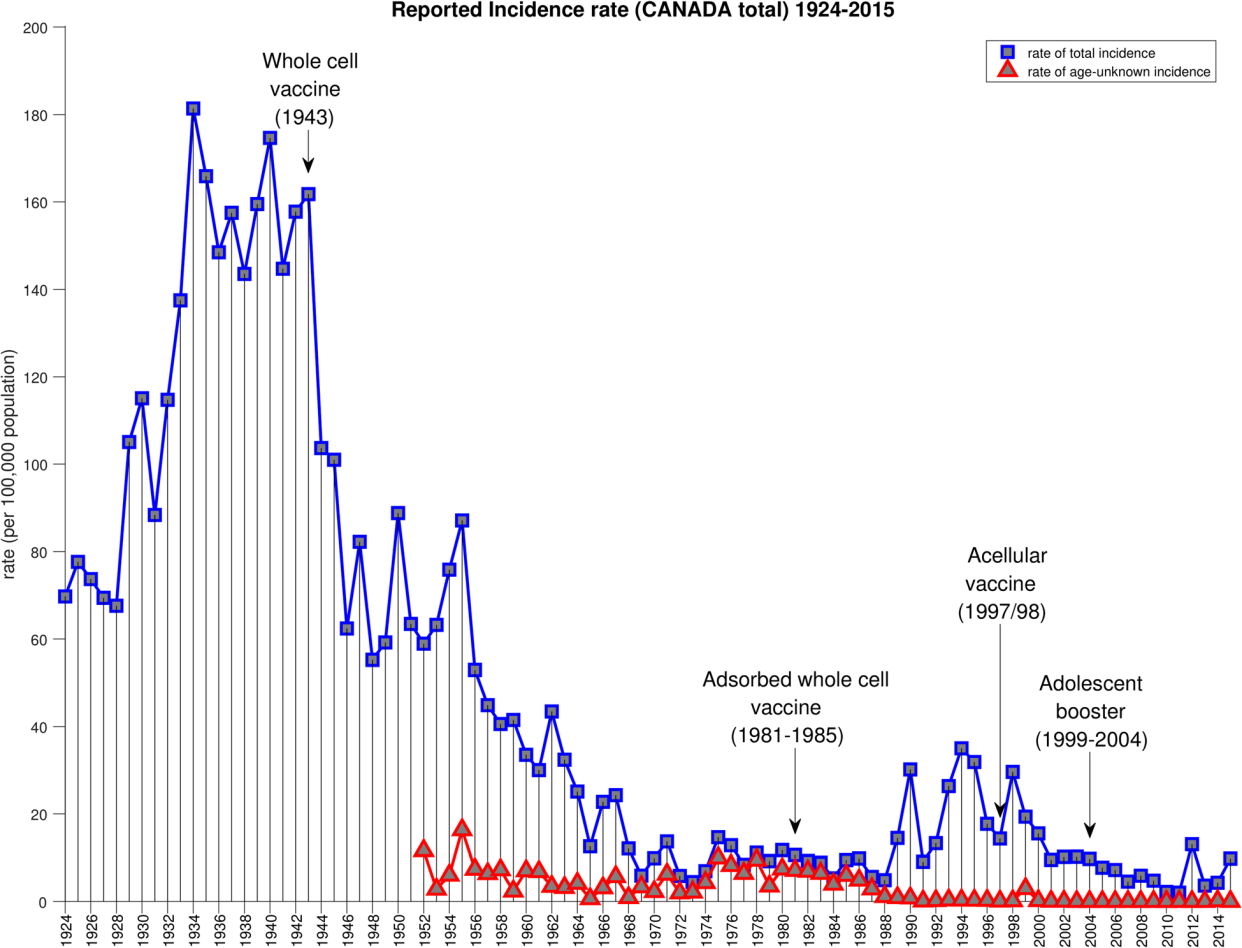
Summary of the current state of knowledge of this history of pertussis epidemiology in Canada: Yearly incidence rates for total reported cases of pertussis since the start of national reporting in 1924 (blue), and yearly incidence of cases without age information (red). Prior to 1952, no age information was reported at all. See Fig. S1 for a more detailed view of the period 1952–2015. In most years between 1969 and 1988, the majority of cases had no age information

Figure 1 depicts the total yearly incidence rates *R*(*i*) together with, from 1952 onward, the age-unknown rate *R*^*U*^(*i*) (since no cases were collected with age information befor 1952, *R*^*U*^(*i*) ≡ *R*(*i*) during this time). As can be seen, the rate of cases reported without age information varied substantially between 1952 and 1989, before becoming more or less uniformly low from 1990 onward, when Ontario completed the transition from partial to full reporting of the age (and sex) of pertussis cases.

From 1952 onward, it has thus been possible to calculate age-stratified rates, albeit incomplete ones:

2 $$ {\hat{R}}_j(i)=\frac{C_j^K(i)}{P_j(i)},i=1952,\cdots, 2015\ \mathrm{and}\ j=1,\cdots, n $$

where subindex *j* denotes the *j* th age group, *n* is the number of age groups, and ${C}_j^K(i)$ is the number of cases reported in age group *j*. It is important to note that ${\hat{R}}_j(i)$ is always a *lower limit* to the true rate *R*_*j*_(*i*), because each age group can also contain additional cases whose ages were not reported.

### Adjustment of rates for missing age information

**Fig. 2:**
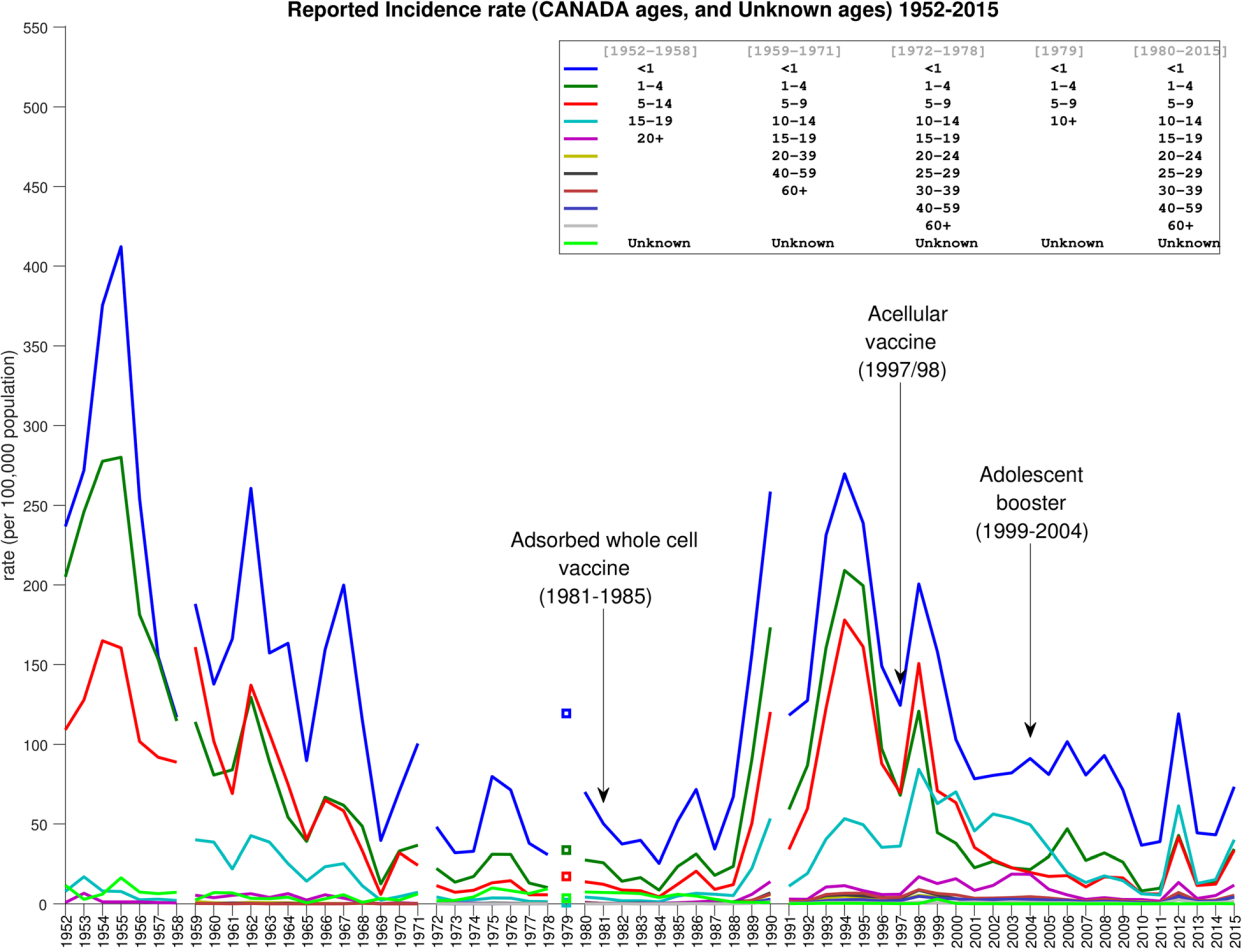
Yearly age-stratified incidence rates for age-supplied reported cases ($\hat{\ {\boldsymbol{R}}_{\boldsymbol{j}}}$) and “Unknown” group $\hat{{\boldsymbol{R}}^{\boldsymbol{U}}}$. Note that some of the age groupings changed during the years of reporting; these changes are denoted by gaps in the graph. Contrary to Fig. 1, the incidence rate for age-unknown cases is comparatively small throughout, since its denominator is the entire population rather than just one age group

Figure 1 shows that from the beginning of age-stratified reporting in 1952, all the way until 1988, there has been substantial variation from year to year in the proportion of the overall incidence rate that is reported without age information. Very importantly, in most years between 1968 and 1988, the majority of cases were reported without ages. Yet, as Fig. 2 shows, *R*^*U*^(*i*) is small compared to the individual age-stratified rates below age 5. This is because the burden of pertussis is concentrated largely below that age, whereas the definition of *R*^*U*^(*i*) amounts to evenly distributing the age-unknown cases across *all* ages. Thus, *R*^*U*^(*i*) can be a misleadingly low indication of how much of the burden of pertussis in young ages is hidden among the age-unknown cases. Likewise, we can infer that variations over time in *R*^*U*^(*i*) have an artificial component driven by the variations in the proportion of age-unknown cases.

Rather than making use of *R*^*U*^(*i*), we develop here an estimate $\overset{\sim }{R_j}(i)$, of the true rate *R*_*j*_(*i*), of pertussis in age group *j*. This consists of making the simplest possible imputation of missing age information: We assume that cases with unknown ages have a similar age distribution as those with reported ages. This assumption comes with a number of caveats, which are considered in the Discussion section below. We obtain (see Appendix A2 for details)

3 $$ \overset{\sim }{R_j}(i)=\frac{C_j^K(i)}{P_j(i)}\times \frac{C(i)}{C^K(i)}={\hat{R}}_j(i)\times \frac{C(i)}{C^K(i)}, $$

where *C*(*i*) is the total number of cases in year *i*, and *C*^*K*^(*i*) is the total number of cases with known ages. In other words, we multiply the lower-limit rates (Eq. 2) by a common correction factor. We also estimated the true rate of pertussis in each age via the bootstrapping method. Using the Kruskal-Wallis test [33], we found there is no statistically significant difference between the estimated $\overset{\sim }{R_j}(i)$ based on formula (3) and the estimated age-stratified true rate using the boostrapping method, see again Appendix A2 for details.

## Results

**Fig. 3:**
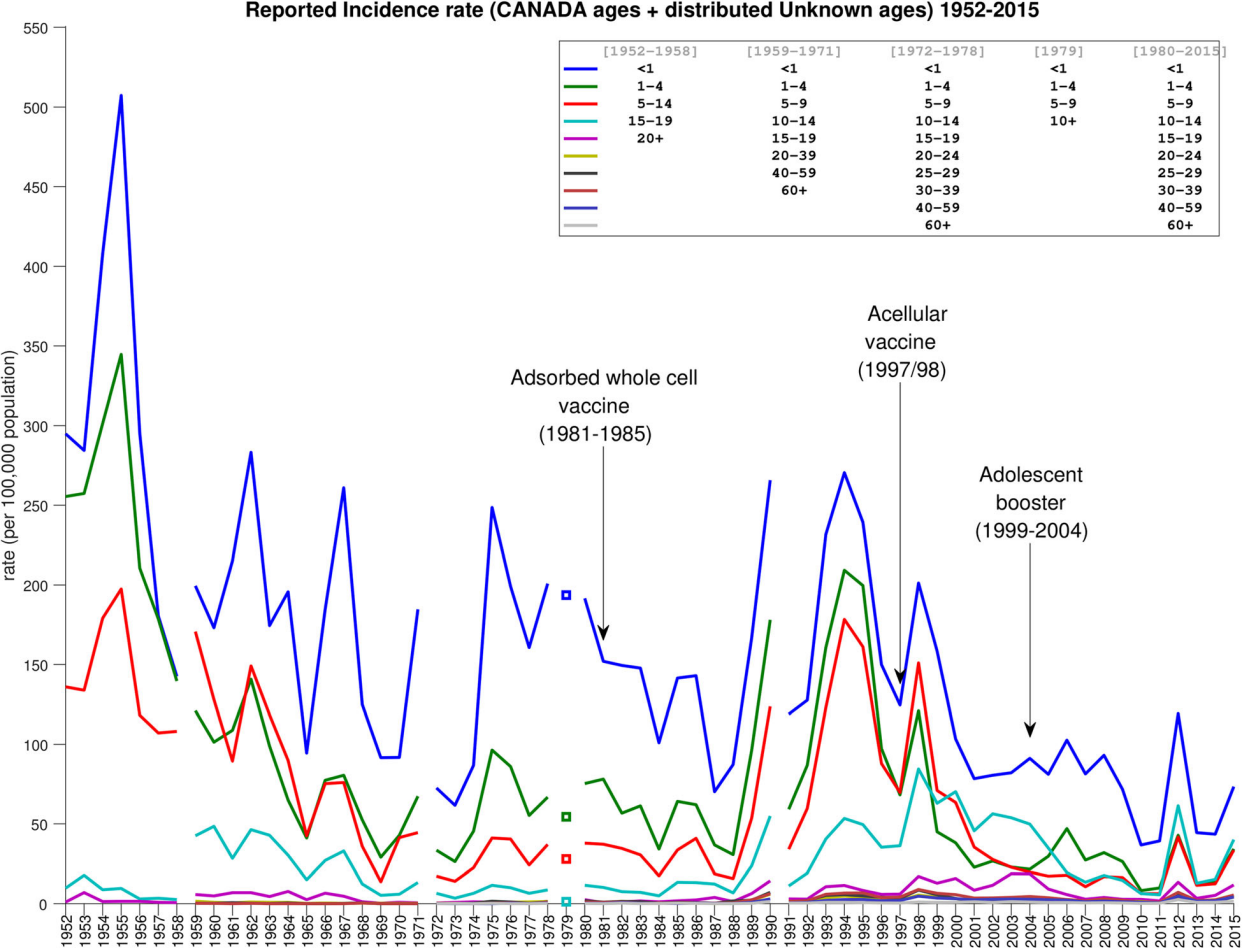
Yearly adjusted age-stratified incidence rates for combined age-supplied reported cases and proportionally-distributed “Unknown” group $\overset{\sim }{\ {\boldsymbol{R}}_{\boldsymbol{j}}}$, also calculated via the bootstrapping method. Note that some of the groupings changed during the years of reporting; these changes are denoted by gaps in the graph

Fig. 3 shows the approximated age-stratified rates from 1952 to 2015. Compared to Fig. 2, two key differences are apparent: First, Fig. 2 shows the lowest incidence rates in pertussis across all ages occurring during the period from about 1969 to about 1987, with a sharp increase beginning in 1988. This outbreak continues to about 1999, and rates during this time are comparable to the period 1952–1968. In contrast, Fig. 3 shows substantially higher rates in the period from 1969 to 1987. As a result, the contrast between pre- and post-1988 incidence is, across all ages, less sharp than in Fig. 2. Secondly, in Fig. 2, for all ages the average yearly incidence in the 2000–2015 period is higher than in the 1969–1987 period. In other words, this implies that after the 1988–1999 outbreak, although rates fell again, they remained above the pre-outbreak level. In contrast, in Fig. 3, the post-outbreak yearly rates are, below age 5, on average slightly lower than they were in the (pre-outbreak) 1969–1987 period. Just as importantly, though, the post-outbreak rates for ages 10 and up are on average significantly higher than the pre-outbreak rates.

## Discussion

As summarized in Section 0, multiple studies of Canadian pertussis surveillance data point to a strong resurgence of pertussis in 1989, widely interpreted as being caused by the adsorbed whole-cell pertussis vaccine (replacing the original whole-cell vaccine) that was first introduced in Canada in 1981, and by 1985 had been fully adopted across the country. As Table 1 shows, it is widely inferred among researchers that this created a cohort of less-well-protected children which, a few years later, fueled a large and abrupt increase in pertussis incidence across all ages.

Numerous researchers have pointed to a worldwide resurgence in pertussis and causally linked this to the switch from whole-cell to acellular vaccine (see [34] and references therein). However, this claim remains controversial; other studies suggest that the rise of incidence after switching to acellular vaccine was not a universal phenomenon, and even that it predated the switch in some countries [34]. In Canada, the epidemiological picture is complicated by the fact that there was an intermediate period of adsorbed whole-cell vaccine use. If one makes causal links from changes in vaccine to subsequent changes in epidemiology, then the uncorrected age-stratified rates (Fig. 2) would seem to indicate that (i) adsorbed whole-cell vaccine was substantially inferior to the original whole-cell vaccine, and (ii) although acellular performed better than adsorbed whole-cell, it failed to drive the incidence rates as low as they had been under the original whole-cell vaccine.

Our adjusted age-stratified rates (Fig. 3) presents a modified picture: First, although they do also show the 1989–1999 outbreak, due to the higher pre-outbreak rates, the jump in rates is less sharp across all ages, especially below age 1. Thus, though our results also suggest that adsorbed whole-cell vaccine performed more poorly than the original whole-cell, the difference may have been less extreme than previously inferred. Furthermore, acellular vaccine may have performed better, not worse, than the original whole-cell below age 5. However, in ages 10 and up, our results show higher incidence rates than during the era of the original whole-cell vaccine, suggesting that protection may have declined in those ages. Additionally, since almost all reported cases have had age information since 1988, corrected and uncorrected rates are near-identical from that year onward. Thus, we see in Fig. 3 the same 2012 spike in incidence rate as in Fig. 2.

It is important to note that the methodology we have presented here for adjusting age-stratified pertussis surveillance rates has limitations despite the validation using the bootstrapping method. Our fundamental assumption is that the age distribution of age-unknown cases is the same as that of cases with reported ages. One potential issue here is that prior to 1988, different Canadian provinces/territories had different guidelines for age reporting of pertussis cases. For this reason, age-unknown cases have tended to be geographically clustered. There is thus the possibility that systematic differences exist between the populations contributing age-reported versus age-unknown cases. This could lead, in turn, to differences in the age distribution. We have not attempted here to quantify how large such deviations would have to be to invalidate our imputation method (in the sense that the corrected rates, $\overset{\sim }{R_j}(i)$ are a worse approximation of the true rates *R*_*j*_(*i*), than are the uncorrected rates ${\hat{R}}_j(i)$). This is an interesting topic for follow-up work and could be addressed using simulated data.

Another important caveat is that for most of the years from 1969 to 1987, the majority of pertussis cases across Canada were reported without age (Fig. 1). This corresponds to the time during which Ontario had started to report pertussis cases but was doing so with no or only partial age information. This means that during this period, we are using a minority of the pertussis cases to extrapolate the age distribution for all the rest. It should be emphasized that we have not attempted to correct for any other aspect of reporting practices aside from the reporting (or not) of ages. In particular, we have not considered the possible effect of changes in test sensitivity or frequency of testing.

## Conclusions

We have synthesized, for the first time, age-stratified incidence rates of pertussis in Canada for a period of more than 60 years (1952–2015) using surveillance data. In doing so, we developed and made use of an extrapolation method that distributes among the age groups those cases collected without age information. Additionally, we reviewed published reports and articles which, during this period, described and studied trends in incidence, and proposed theories for the underlying causal factors. Our results cast new light on parts of the history of pertussis in Canada, and suggest that some causal inferences drawn about changes in pertussis epidemiology over time may need to be revisited. There is still no full consensus of interpretation of the temporal trends of pertussis epidemiology in Canada. A critical and first step towards a consensus is to have accurate age-specific information for these temporal trends to help in parameterizing age-specific transmission dynamics models with contact mixing in order to reduce variables and identify key drivers for the variation of incidence data between outbreaks and between interepidemic periods. We hope that this work contributes to the ongoing effort to understand more fully the dynamics of pertussis in Canada.

## Supplementary Information

**Additional file 1: **Wu-IRC-pertussis-supp-BMCPH-June10.pdf. “Supplementary Material”. Supplementary material, comprising sections A1, A2 and A3. **Table S1**. References to data sources accessed/consulted for different sub-periods of 1952–2015 for age stratified and 1924–2015 for total reported incidences. **Table S1**. References to data sources accessed/consulted for different sub-periods of 1952–2015 for age stratified and 1924–2015 for total reported incidences. **Table S1**. References to data sources accessed/consulted for different sub-periods of 1952–2015 for age stratified and 1924–2015 for total reported incidences. **Fig. S1**. Yearly incidence rates for total *R* (blue) and age-unknown ${\hat{R}}^u$ (green) reported cases of pertussis since the start of national age-stratified reporting in 1952. **Fig. S2**. Comparison of age-stratified distributions calculated in three different ways: 1) age distribution that assuming all cases have the same age distribution as those ageknown cases (*R1*); 2) age distribution estimated by the bootstrapping method (*R2*); 3) age distribution of those age-known cases only (*R3*). An application of the Kruskal-Wallis test shows no statistically significant difference among these distributions. **Fig. S1**. Yearly adjusted age-stratified incidence rates for combined age-supplied reported cases calculated via the bootstrapping method. **Fig. S4** The age-stratified proportions (with respect to total) for each of the four provinces Ontario, Quebec, British Columbia, and Alberta in years of 1991, 1992, 1993, and 1994.

## Data Availability

All data relevant to the study are included in the article or uploaded as supplementary information.
