# Supplementary material for: Revisiting the epidemiology of pertussis in Canada, 1924–2015: a literature review, evidence synthesis, and modeling study
Source: BMC Public Health. 2020 Nov 20;20:1749. doi: 10.1186/s12889-020-09854-4 (PMC7678223; doi:10.1186/s12889-020-09854-4)
Supplement: Supplementary file 1 — Additional file 1: Wu-IRC-pertussis-supp-BMCPH-June10.pdf. “Supplementary Material”. Supplementary material, comprising sections A1, A2 and A3. Table S1. References to data sources accessed/consulted for different sub-periods of 1952–2015 for age stratified and 1924–2015 for total reported incidences. Table S1. References to data sources accessed/consulted for different sub-periods of 1952–2015 for age stratified and 1924–2015 for total reported incidences. Table S1. References to data sources accessed/consulted for different sub-periods of 1952–2015 for age stratified and 1924–2015 for total reported incidences. Fig. S1. Yearly incidence rates for total R (blue) and age-unknown ${\hat{R}}^u$R^u (green) reported cases of pertussis since the start of national age-stratified reporting in 1952. Fig. S2. Comparison of age-stratified distributions calculated in three different ways: 1) age distribution that assuming all cases have the same age distribution as those ageknown cases (R1); 2) age distribution estimated by the bootstrapping method (R2); 3) age distribution of those age-known cases only (R3). An application of the Kruskal-Wallis test shows no statistically significant difference among these distributions. Fig. S1. Yearly adjusted age-stratified incidence rates for combined age-supplied reported cases calculated via the bootstrapping method. Fig. S4 The age-stratified proportions (with respect to total) for each of the four provinces Ontario, Quebec, British Columbia, and Alberta in years of 1991, 1992, 1993, and 1994. [file 12889_2020_9854_MOESM1_ESM.pdf]

Supplementary Material

Revisiting the epidemiology of pertussis in Canada, 1924-2015: A literature review, evidence synthesis, and modeling study

Edward Thommes, PhD[1,2,3], Jianhong Wu, PhD[3], Yanyu Xiao, PhD[4], Antigona Tomovici, MD[1], Jason Lee, MSc, MBiotech[1,5], Ayman Chit, PhD, MBiotech[1,5]

A1. Details of Case Report Implementation by Provinces and Territories

Tables S1-S3 give details of the reports and references used for our analysis. Table S1 lists the resources which are usually annual reports with tabulated data, except for 1979 which is graphical data. For this year the data for age groups <1y, 1-4y, and 5-9y are taken from the graphs and the data for >=10y is estimated by first calculating the total from the information on relative ratio of <10y (the sum of <1y, 1-4y, and 5-9y) over the total, and then subtracting the total from <10y. Table S2 lists some details of the gradual implementation of national reporting by provinces/territories, and Table S3 lists similar details involving age/sex information.

Table S1: References to data sources accessed/consulted for different sub-periods of 1952-2015 for age stratified and 1924-2015 for total reported incidences.

Years	Source of data
1991-2015	National Notifiable Diseases [3]
1980-1990	Smith et al. [4], Figure 3; Personal communication with authors
1979	Varughese [10], Figure 4 (part of 1960-1984, <1 y,1-4 y, 5-9 y)<br>Varughese [10], Figure 3 used for calculation of >= 10 y
1972-1978	Annual Report of Notifiable Diseases [8]
1959-1971	1971, Annual Report of Notifiable Diseases [8]<br>1961-1970, Annual Report of Notifiable Diseases [7]<br>1959-1960, Annual Report of Notifiable Diseases [6]
1952-1958	Annual Report of Notifiable Diseases [6]
1924-2015	National Notifiable Diseases [3], used for total

Table S2: National reporting of total (regardless of age/sex) started in 1924 and gradually implemented in all provinces/territories.

Years	Provinces/Territories Not Reporting
1924-1928	Prince Edward Island, Newfoundland, Yukon, Northwest Territories
1929-1949	Newfoundland, Yukon, Northwest Territories
1950-1955	Yukon, Northwest Territories
1956-1958	Northwest Territories
1959	-
1960-1962	Northwest Territories
1963-2015	-
Notes: Nunavut data were partial and incomplete for most years.	

Table S3: National government encouraged reporting of age/sex around 1952 and provinces/territories gradually adapted the system during the coming decades.

**Years**	Newfoundland and Labrador	New Brunswick	Manitoba	Saskatchewan	Alberta	British Columbia	Yukon	Northwest Territories	Quebec	Prince Edward Island	Nova Scotia	Ontario	Nunavut
1952-1959	X	X	X	X	X	X							
1960	X	X	X	X	X	X	X						
1961-1963	X	X	X	X	X	X							
1964-1965	X	X	X	X	X	X	X						
1966-1968	X	X	X	X	X	X		X					
1969-1970	X	X	X	X	X	X	X	X					
1971-1978	X	X	X	X	X	X	X	X	X	N	N	N	
1979-1987	X	X	X	X	X	X	X	X	X	P	P	P	
1988-1989	X	X	X	X	X	X	X	X	X	X	X	P	
1990	X	X	X	X	P	X	X	X	X	X	X	X	
1991-1998	X	X	X	X	X	X	X	X	X	X	X	X	
1999	X	X	X	X	X	X	X	P	X	X	X	X	P
2000-2015	X	X	X	X	X	X	X	X	X	X	X	X	X

Notes: Nunavut data were partial and incomplete for most years. "X" indicates reporting age and sex for the majority of cases. "N" indicates not reporting age or sex. "P" indicates partial (incomplete) reporting of age or sex. There were many instances where age was not stated but sex was stated for some cases. However, in cases where sex was not stated, age was not stated either. From 1952 to 1970, and from 1991 onward (with the exception of the Northwest Territories and Nunavut in 1999), all provinces that reported pertussis cases reported the majority of them with ages. The largest proportion of age-unknown cases occurred in the period from 1971 to 1989, when there was no or partial age reporting in Ontario. This can also be seen in Figure S1 below.

A2. Adjustment of Incidence Rates for Missing/Incomplete Reports

Consider a population divided into n age groups, such that

$$P = P_1 + P_2 + \cdots + P_j + \cdots + P_n$$

where P_n is the population sizes in age group n. Suppose we are provided with incidence data for year i, for each age group:

$$C = C_1 + C_2 + \cdots + C_j + \cdots + C_n$$

where C_1, C_2, C_j and C_n are the number of cases in age groups 1, 2, j and n, respectively, and we have dropped the year index i for convenience.

We can further divide the above into

$C_j = C_j^K + C_j^U$

C_j^K	number of cases in age group j for which age was reported ("Known")
C_j^U	number of cases in age group j for which age was not reported ("Unknown")

In other words, in reality we only know the total number of cases C, and the age-known cases C_j^K in each age group j (and implicitly $C^K = C_1^K + \cdots + C_n^K$, and hence $C^U = C - C^K$).

The true values of the incidence in each age group are

$$R_j = \frac{C_j}{P_j}, \qquad j = 1, \ldots n$$

These are not computable because the C_j^U, and thus the C_j, are not known. Here we develop a method to approximate these values.

The underlying assumption we make is that the relative age distribution of the unknown cases is approximately same as that of the known cases:

$$\frac{C_j^U}{C_U} \approx \frac{C_j^K}{C_K}, \qquad j = 1, \dots n$$

Thus, our approximations (which we denote with a tilde) for the unknown incidences are

$$\widetilde{C_j^U} = C_j^K \times \frac{C^U}{C^K}, \qquad j = 1, \dots n,$$

and hence our approximations for the true incidences are

$$\widetilde{C_j} = C_j^K + \widetilde{C_j^U} = C_j^K + C_j^K \times \frac{C^U}{C^K} = C_j^K \times \left(1 + \frac{C^U}{C^K}\right) = C_j^K \times \left(\frac{C^K + C^U}{C^K}\right) = C_j^K \times \frac{C}{C^K}$$

Thus our approximations for the incidence rates are

$$\widetilde{R_J} = \frac{\widetilde{C_J}}{P_j} = \frac{C_j^K}{P_j} \times \frac{C}{C^K} \qquad (4)$$

A number of publications have reported incidence values cases with known and unknown ages separately. Their approximations (which we denote with a hat) for the rates in each age group *j* are

$$\widehat{R_J} = \frac{C_j^K}{P_j} \qquad (7)$$

They then additionally report an overall "age-unknown rate",

$$R^U = \frac{C^U}{P} \qquad (8)$$

Since each age group can have cases that are age-unknown and thus uncounted in that group, the $\widehat{R_J}$ are *minimum* rates. R^U gives an overall measure of how much underestimation is taking place. However, it is important to note that the definition of R^U amounts to evenly distributing the age-unknown cases across all ages, when in fact the burden of pertussis is concentrated almost entirely below age 20. Thus, when comparing to the $\widehat{R_J}$ of a given age group, R^U can be a misleadingly low indication of how much of the burden of pertussis is contributed by age-unknown cases.

Figure S1 shows the yearly rate across all ages, $R = C/P$, as well as the age-unknown rate R^U. We note that especially during the years 1965-1987, the large relative value of R^U suggests that it is necessary to interpret unadjusted, age-stratified rates with great caution.

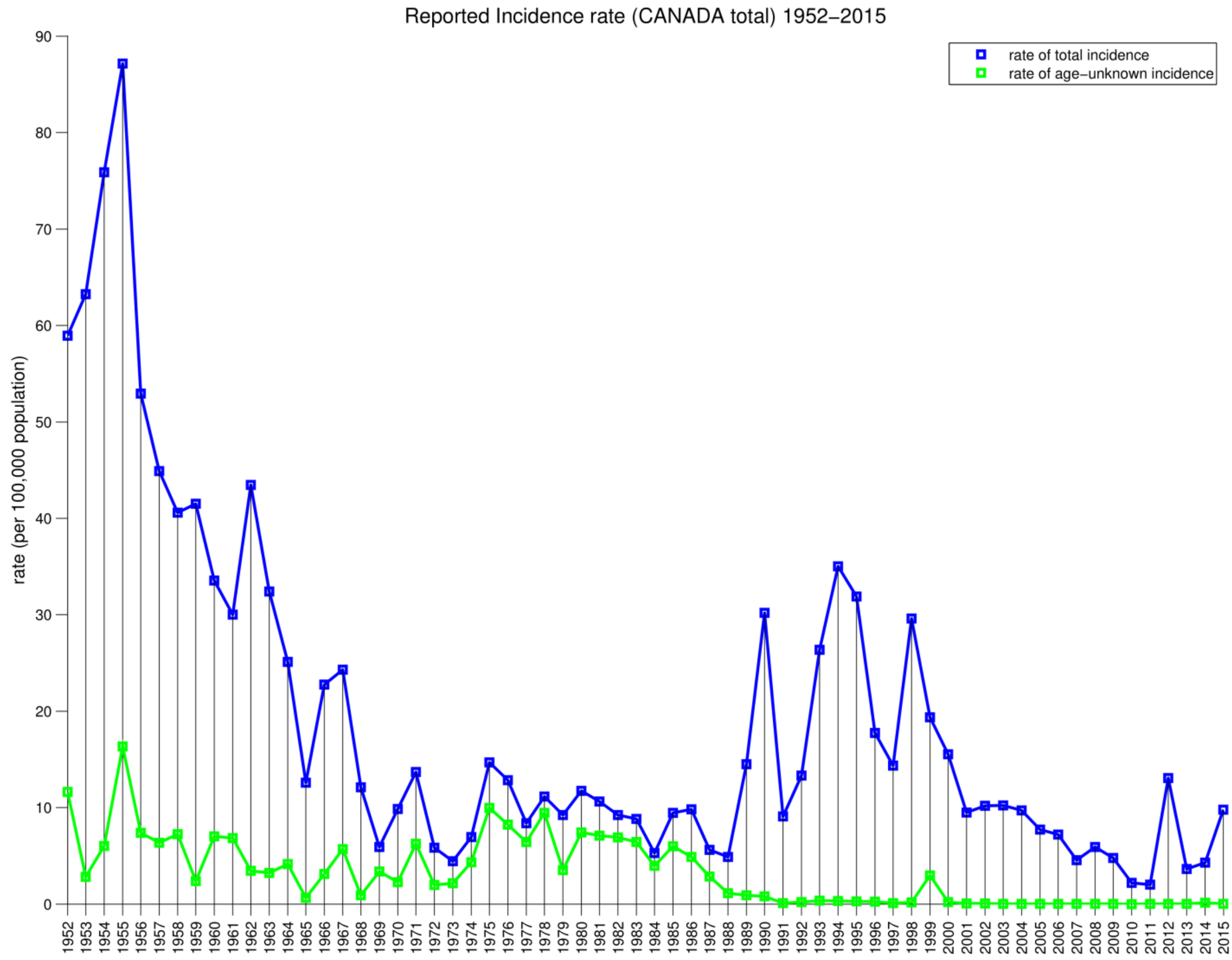

Figure S1: Yearly incidence rates for total R (blue) and age-unknown $\widehat{R^U}$ (green) reported cases of pertussis since the start of national age-stratified reporting in 1952.

We also applied the bootstrapping method to verify these approximate age-stratified incidence rates. Under the assumption that the relative age distribution of the unknown cases is approximately same as that of the known cases, the approximations (which we denote with a tilde) for the unknown incidences are

$$\tilde{C}_j^U(i) = C^U \times \ D_j^{KS}(i)\,, \qquad j = 1, \dots n,$$

with $D_j^{KS}(i)$ being the calculated age-stratified distribution for age group *j* in year *i* via the bootstrapping method from C^K. We iteratively resampled with replacements from $C^K(i)$ 200

times to get a dataset of the same size of $C^U(i)$. Then we calculated the age distribution for each dataset and used the average of them to estimate the age distribution of $C^U(i)$. The approximations for the incidence rates are

$$\tilde{R}_j = \frac{C_j^K(i) + \tilde{C}_j^U(i)}{P_j(i)} = \frac{C_j^K(i) + C^U(i) \times D_j^{KS}(i)}{P_j(i)}$$

In Figure S2, four years (1972, 1980, 1985 and 1991) of age-stratified total incidences with age-unknown cased being assigned to age groups following the distribution $D_j^{KS}(i)$ are presented. In order to validate our estimated age distributions $D_j^{KS}(i)$, we also compared them with the age distribution of age-known cases (see Figure S3) for these four years, respectively. In addition, we calculated the age distribution of incidence during these years by assuming that all cases including unknown ages have the same age distribution as those with reported ages in Figure S2. We observed that distributions we calculated in three different approaches are almost identical to each other. Applying the Kruskal-Wallis test, we found there is no statistically significant difference among these distributions (all $p<0.05$).

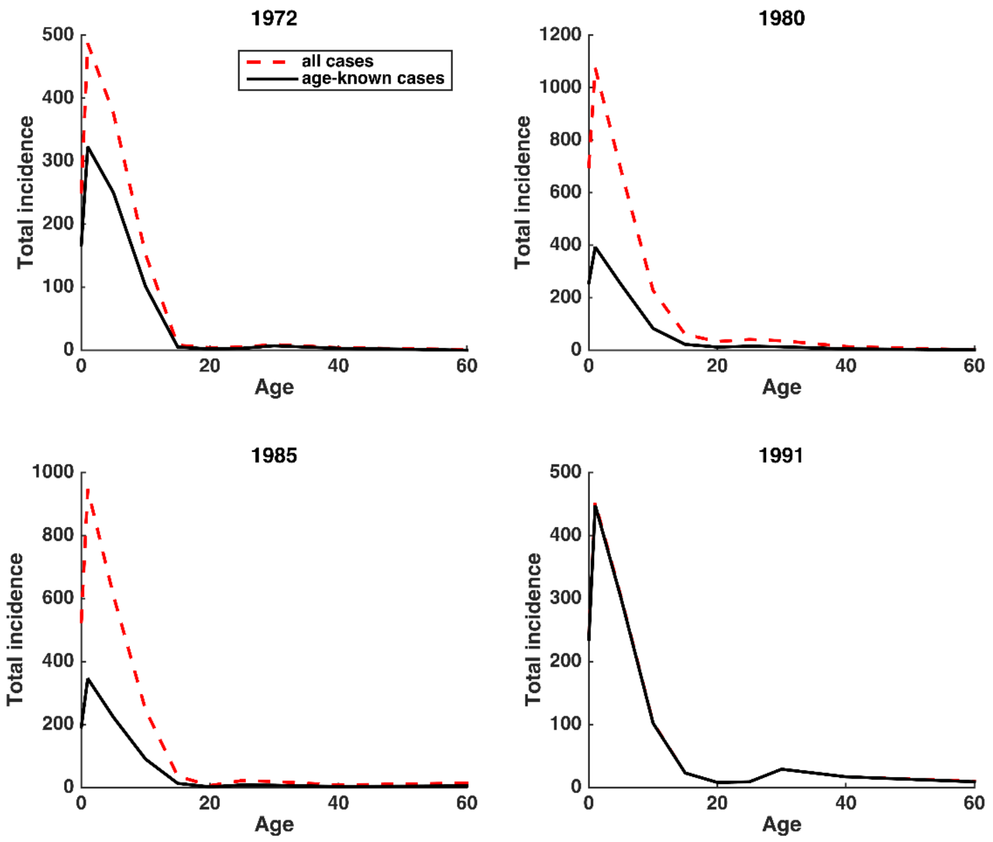

Figure S2 shows the comparison of three age distributions: 1) age distribution that assuming all cases have the same age distribution as those age-known cases (R_1); 2) age distribution estimated by the bootstrapping method (R_2); 3) age distribution of age-known cases only (R_3).

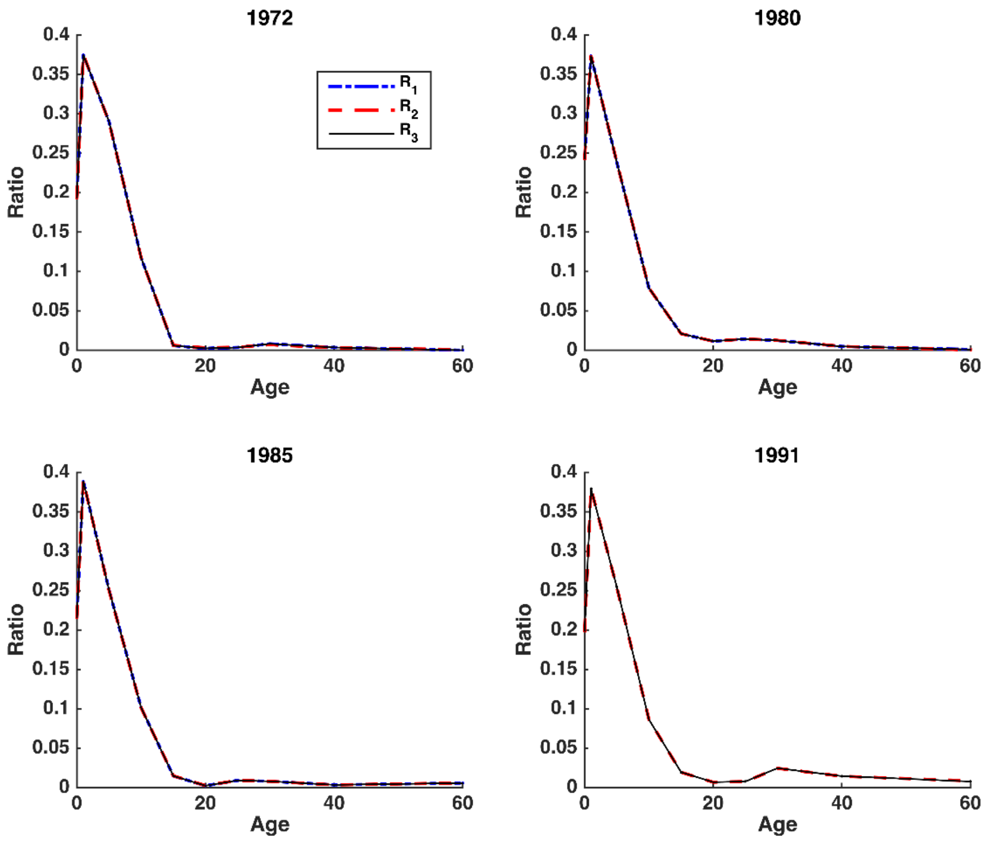

Figure S2: Comparison of age-stratified distributions calculated in three different ways: 1) age distribution that assuming all cases have the same age distribution as those age-known cases (R_1); 2) age distribution estimated by the bootstrapping method (R_2); 3) age distribution of those age-known cases only (R_3). An application of the Kruskal-Wallis test shows no statistically significant difference among these distributions.

It is thus natural that the yearly adjusted age-stratified incidence rates for combined age-supplied reported cases, calculated via the bootstrapping method and shown in Figure S3, remains essentially the same as Figure 3.

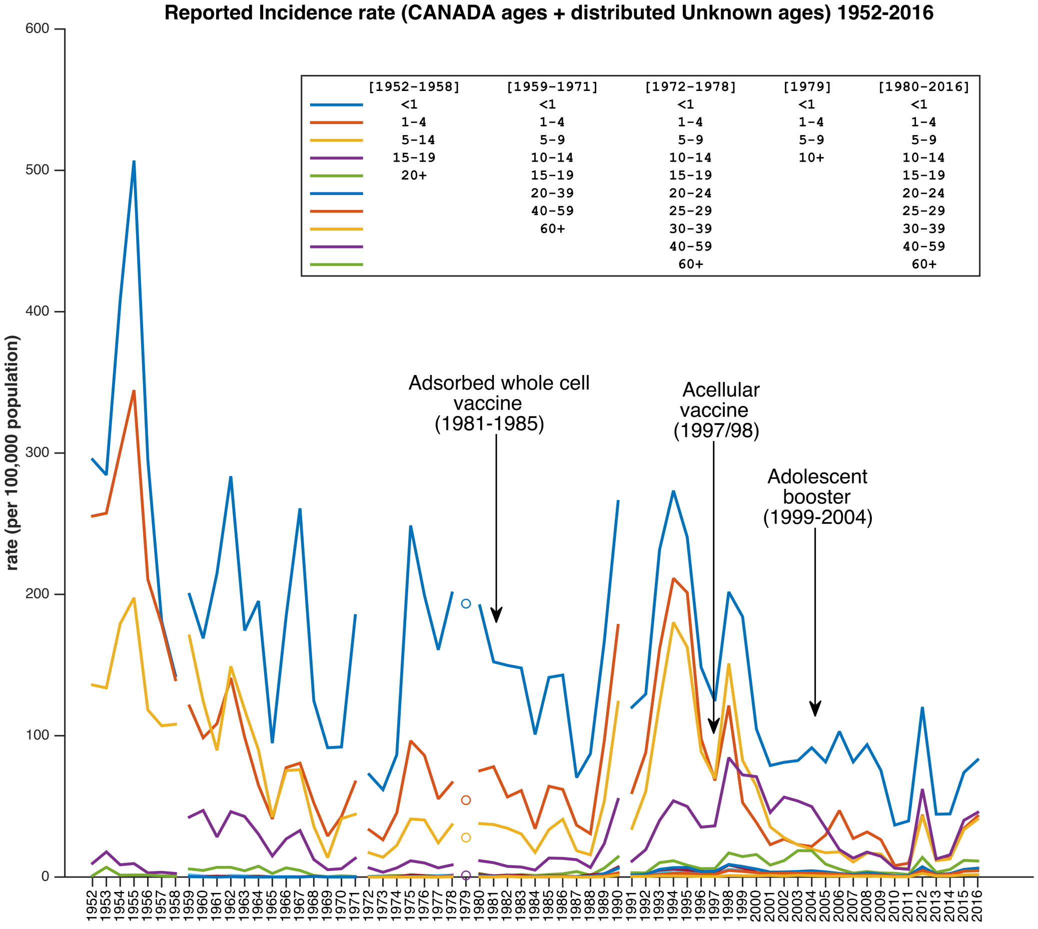

Figure S1: Yearly adjusted age-stratified incidence rates for combined age-supplied reported cases calculated via the bootstrapping method.

We were able to extract from the incidence data for the four provinces Ontario, Quebec, British Columbia, and Alberta for each of the years 1991, 1992, 1993, and 1994, the age-stratified incidences, as these provinces reported ages for the majority of cases. Using these age-stratified incidences, we calculated the age-stratified proportions (with respect to total) for each province. As shown in Figure S4, the patterns for Ontario were comparable to the other three provinces, showing the existence of a Canadian trend which each province follows approximately.

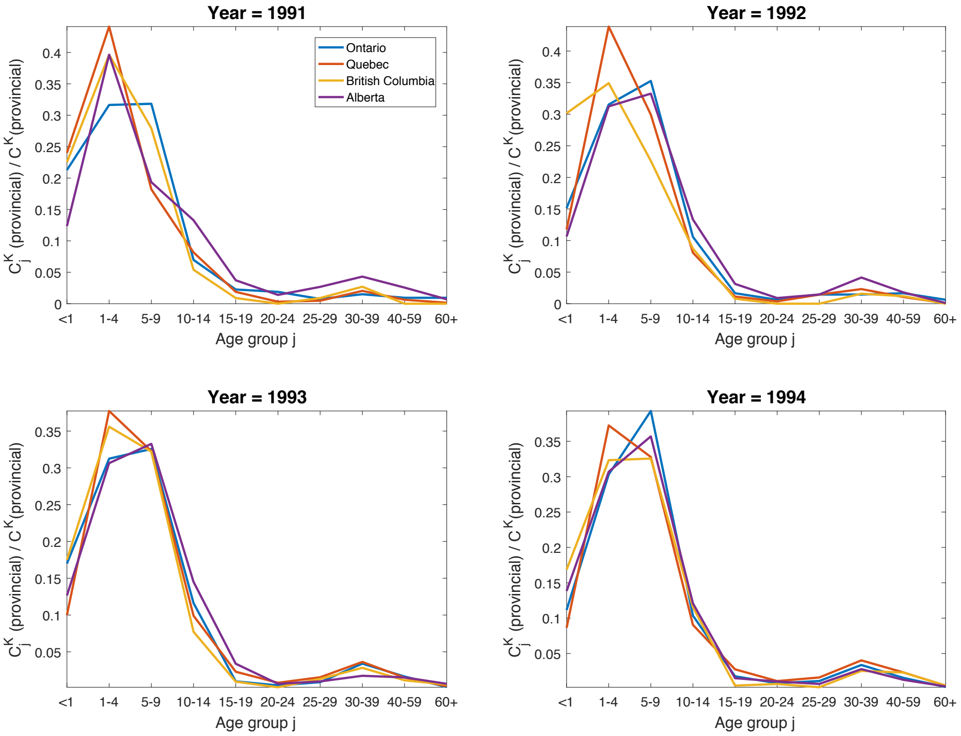

Figure S4: The age-stratified proportions (with respect to total) for each of the four provinces Ontario, Quebec, British Columbia, and Alberta in years of 1991, 1992, 1993, and 1994.

Age-stratified incidences could be calculated since these provinces reported ages for the majority cases. The figure shows that the patterns for Ontario were comparable to the other three provinces, showing the existence of a Canadian trend which each province follows approximately.

A3. Details of Reports/Articles Presenting and/or Interpreting Incidence in Canada

- Ross [1] reported on mortality rates for the period 1880-1929 of pertussis and three other infectious diseases in Ontario. The paper made comparisons from different aspects including age, sex, and type of residence (urban vs. rural), and also some inter disease cross comparison, to discuss possible hypotheses underlying the disease dynamics. Interestingly, the paper warned about seemingly plausible explanations that only prove inconsistent with certain comparisons.
- Data presented in the Museum of Health Care [2], extended the previous mortality data to 1934 and also showed the morbidity data for the period 1905-1934. It mentioned that even though pertussis was a reportable disease in the province, almost no apparent usage came from collected data and no action was taken in response to the epidemics.
- The first published article after the introduction of immunization reported the total incidence for the period 1924-1978 along with the death rates [5]. This also included age stratified incidence data for 1960-1978 and some hospitalization statistics for 1969-1976. The article mentioned general decline after vaccine introduction in line with results from field trials in Quebec and Alberta during 1943-1945 with more than 90% reported effectiveness [24], [25]. However, this descriptive report was cautious to interpret the trends solely based on the effect of the vaccine, mentioning other changes in living and societal conditions that might have affected the epidemiology.
- Later, a similar study extended the reporting period to 1924-1984 [10]. This study made an interesting observation that hospitalization rates and incidence rates were almost equal for years close to the end of the reporting period, which the study

attributed to incompleteness of the incidence reports. However, an assumed correction factor of 50% was used with little justification.

- The study by Halperin et al. [11] looked into the alarming high rates of pertussis in Nova Scotia (compared to Canada as whole and the USA) by conducting a 28-month study around mid-1980s in Nova Scotia involving the use of an enhanced pertussis surveillance and laboratory diagnosis. They conducted this study in three phases with varying degrees of case detection effort and during periods of different endemicity. They then, concluded that the choice of surveillance and confirmation methods has a significant effect on observed incidence records and visible epidemiology. Observing the uniform age-specific distribution of cases for children <10y which resembled patterns from pre-vaccine era, this study added to the rising concerns related to the poor effectiveness of the adsorbed whole-cell vaccine used at the time.
- Skowronski et al. [12] examined the age stratified incidence during 1981-2000 in British Colombia. This study mentioned a number of factors, including: the introduction of PCR, the switch to a poor adsorbed whole-cell vaccine and the resulted cohort effect, the subsequent switch to a more effective acellular vaccine, and the possibility of waning of the protection from vaccines, as the factors that most probably influenced the observed change in epidemiology. Galanis et al. [26] challenged the way of Skowronski et al. [12] that was used to generalize their observations and conclusions to the whole of Canada, arguing that there was no evidence of synchronized epidemiology. Skowronski et al. [27] responded by claiming that even though the patterns in different provinces may not be the same in a specific year, this was only because the poorly protective adsorbed whole cell was introduced in different years in different provinces.

- Later on, Ntezayabo et al. [13] analyzed the incidence and hospitalization data between 1983 and 1998 from Quebec and concluded that the cohort effect was present. They also pointed to the possible role of improved surveillance and reporting by further comparing hospitalization rates and incidence rates, and noting no apparent other explanations for the change of their ratio through time.
- Galanis et al. [14] used Canadian incidence data 1924-2002, age stratified for 1988-2002 to suggest that switch to acellular vaccines reversed the observed resurgence of the 1990's for small children. This study suggested that at that time the recently introduced adolescent booster would have similar effect for age group 15-19y. Authors expressed their concern about reduced natural boosting as a result of increased vaccination with subsequent waning of vaccine immunity, creating a pool of susceptible individuals. This argument was backed by their cohort argument for 1990s recipients of adsorbed whole-cell vaccine. They, then, mentioned that a single dose adult booster (as suggested by the National Advisory Committee on Immunization) would protect adults against infection and reduce transmission from adults to their contacts.
- Vickers et al. [15] used the passive surveillance data from the Saskatoon Regional Health Authority in Saskatchewan for the period 1995–2005 to assess the transition from adsorbed whole-cell to acellular pertussis vaccine. They used age-stratified data for <10y and compared this with vaccination type and concluded that the adsorbed whole-cell vaccine, or a combination of adsorbed whole-cell and acellular, provided better protection than acellular alone. This conclusion seemed to be in agreement with reports from the US, but was (considered as) surprising for Canada which had used an adsorbed whole-cell with reported low-effectiveness.

- Halperin et al. [28] challenged the claims of Vickers et al. [15] by employing more detailed data from the same place, including >10y age-groups. They showed that the conclusions of Vickers et al. [15] could have been different if they had considered the role of transmission from adolescents and adults, given the increasing reported incidences in these age groups. They also showed that the analysis in Vickers et al. [15] was potentially affected by the timing of outbreaks in Saskatchewan which was different from most other parts of Canada.
- Bettinger et al. [16] analyzed the hospitalizations during 1991-2004 and used the Canadian Immunization Monitoring Program, Active (IMPACT) pertussis database to assess the effect of changing from whole-cell to acellular pertussis vaccine on the characteristics of hospitalized cases of pertussis. Their analysis showed that direct protection gained by acellular vaccine exceeded that of the replaced whole cell. However, the expected indirectly gained herd-immunity for susceptible unvaccinated or partially vaccinated infants <1y was not observed to be much different and the incidence in this age group continued to be high. They suggested the consideration of 1 dose of acellular booster during adolescence or adulthood, as recommended by NACI at the time, to reinforce indirect protection.
- Greenberg et al. [17] looked into age-specific rates of pertussis in Canada, 1988-2004 and the annual number of hospitalized cases of pertussis, IMPACT Centers, 1991-2006, and summarized epidemiological reports. They assessed the effectiveness of combined DTap-Hib and also adolescent/adult Tdap booster. They also reviewed Canadian-relevant publications reporting changes in epidemiology before and after adoption of pertussis/Hib vaccines, and their review found that most of these publications pointed to the steady control of disease in children using DTap-Hib and marked reduction of reported incidence in adolescents and adults in jurisdictions after

introduction of Tdap. They suggested that these vaccination strategies, if adopted, would produce similar benefits as observed in USA.

- Fisman et al. [18] evaluated pertussis trends in the Greater Toronto Area, by analyzing pertussis culture and PCR test records from 1993 to 2007, to inform the control for changing testing patterns and practices. They concluded that the observed size of outbreaks could be magnified by increasing use of testing and improving test sensitivity over time. This study proposed a feedback model to control for bi-directional loop effects, whereby increasing test positivity (due to either increasing incidence or increased test sensitivity) led to increased test submissions by clinicians. They also commented on possible shifted observed seasonality caused by the rise in pertussis testing for undifferentiated cough illness due to wintertime respiratory viruses, a phenomenon that emphasizes the role of diagnosis practice in perceived epidemiology.
- Smith et al. [4] attempted to explain the rising of incidence reported in different degrees from provinces and territories of Canada. They looked at the total incidence data 1924-2012 and age specified incidence 1980-2012. They compared the data with the hospitalization data from IMPACT, 1991-2012, and the Canadian Institute for Health Information's (CIHI) Hospital Morbidity Database (HMD), 1991-2011. The study identified new vaccine introduction or a switch to a more effective vaccine as the reason for decreasing trends. But this study did not address the issue why there is a rise in 2012 and why the hospitalization data did not follow this rise. The authors did present the data by age groups, along with the data for the unknown group (similar to Figure 2 in the main text), but for reasons we discussed in the main text, it is difficult to interpret this study and many discussions may be subject to misinterpretations. This study, however, concluded that for a better understanding of

the transmission dynamics, more accurate reporting, such as vaccination history and bacterial strain characteristics, is needed. This type of data was reproduced in the WHO report [29], which suggested that adolescent booster has been effective, but probably offered too late in life.

- Chambers et al. [19] performed an in-depth analysis of aggregate and age-specific incidence in British Columbia, 1993-2013. This study discussed the underlying reasons for different trends. Similar to previous work in BC [12] and Canada [4] , most of the discussion in Chamners et al. [19] focused on efficacy and waning of vaccines and the effect of their introductions. The study also reported on the ratio of positive tests to overall tests taken which did not show much variation even through the outbreak, highlighting possible effect of improved reporting. But the study mentioned that this possible effect was not always in alignment with the observed trends. In addition, different parts of province showed time and/or intensity variations in observed trends. Overall the study concluded that further monitoring and evaluation is needed for informed decision making based on the trends.
- Responding to the rising incidence reports from several regions in New Brunswick in January 2012, a province-wide outbreak was declared in February [20]. Enhanced age-specific incidence was collected and comparison to baseline incidence (5 year rolling average) rates was used to inform the announcement of the end of outbreak in January 2013. Age-groups 10-14 had the highest incidence rates, and based on suspected waning, were offered a Tdap booster for two regions early in the outbreak, followed by province-wide booster in the fall. Also, catch-up campaigns and booster (Tdap) immunization were recommended for contacts of vulnerable persons and specifically infants. Differences in magnitudes of outbreak and variations in age-

specific incidences between regions were observed, but neither was explained. It was recommended that more studies were required to explain the epidemiology.

- Deeks et al.[21] investigated the emergence and spread of a province-wide outbreak in southwestern Ontario in the period 2011-2013. The work described the outbreak as being initiated in a religious community, then having spread to the general community, and consequently affecting another religious community. The causative description of the study was based on the timing of events. The work's findings showed that the disease profile was different in the under-immunized religious communities from the profile observed in the general community. The former was similar to pre-vaccine trends with most cases in children less than 10 years, and the latter mostly concentrated around pre-adolescent 10-14 years and a non-negligible re-appearance in adults. The study expressed the concern for high proportion of fully immunized cases, especially among 10-14 years age group and called it a warrant for suggestive waning of immunity associated with acellular pertussis vaccine.
- Liu et al. [22] combined records from surveillance data with immunization data to present detailed stratification based on age, sex and geographic location of cases over the years 2004-2015 in Alberta. Based on an analysis which compares with baseline average incidence, the study identified outbreak years. The study reported the observation on variations over the years of incidence as well as differences observed in different geographical areas in the province. The study's conclusions include a possible rising of incidence in general population, which the study believes, was related to the rising reported in other parts of Canada and in the USA. Emphasizing the steadiness of coverage in those years, the study mentioned some potential reasons for its observations but unfortunately did not go further to offer a conclusive argument.

References

1. Ross MA. The Mortality in Ontario of Four Communicable Diseases of Childhood. Can Public Heal J [Internet]. 1932;23(7):331–41. Available from: http://www.jstor.org/stable/41976675
2. Museum of Health Care at Kingston. Pertussis incidence in Ontario, 1880-1934 and 1905-1934. Kingston. C2020
3. Public Health Agency of Canada. Notifiable diseases on-line [Internet]. Available from: https://diseases.canada.ca/notifiable/
4. Smith T, Rotondo J, Desai S, Deehan H. Pertussis Surveillance in Canada: Trends to 2012. Canada Commun Dis Rep. 2014;40(3):21–30.
5. Varughese P, Acres SE. Pertussis in Canada 1924-1978. Canada Dis. Wkly. Rep. 1979; 5(48):217-224.
6. Public Health Agency of Canada. Rate per 100,000 of reported cases over time in Canada, grouped by disease 1952-1960 [Internet]. Reported cases from 1924 to 2017 in Canada - Notifiable diseases on-line. Available from: https://dsol-smed.phac-aspc.gc.ca/notifiable/charts?c=pl
7. Public Health Agency of Canada. Rate per 100,000 of reported cases over time in Canada, grouped by disease 1961-1970 [Internet]. Reported cases from 1924 to 2017 in Canada - Notifiable diseases on-line. Available from: https://dsol-smed.phac-aspc.gc.ca/notifiable/charts?c=pl
8. Public Health Agency of Canada. Rate per 100,000 of reported cases over time in Canada, grouped by disease 1971-1978 [Internet]. Reported cases from 1924 to 2017 in Canada - Notifiable diseases on-line. Available from: https://dsol-smed.phac-aspc.gc.ca/notifiable/charts?c=pl
9. Statistics Canada. Population and demography statistics [Internet]. Available from: https://www.statcan.gc.ca/eng/subjects-start/population_and_demography
10. Varughese P. Pertussis incidence in Canada. Canada Dis Wkly Rep.1985;11(9).
11. Halperin SA, Bortolussi R, MacLean D, Chisholm N. Persistence of pertussis in an immunized population: Results of the Nova Scotia Enhanced Pertussis Surveillance Program. J Pediatr. 1989;115(5 PART 1):686–93.
12. Skowronski DM, De Serres G, MacDonald D, Wu W, Shaw C, Macnabb J, et al. The Changing Age and Seasonal Profile of Pertussis in Canada. J Infect Dis. 2002;185(10):1448–53.
13. Ntezayabo B, De Serres G, Duval B. Pertussis resurgence in Canada largely caused by a cohort effect. Pediatr Infect Dis J. 2003;22(1):22–7.
14. Galanis E, King AS, Varughese P, Halperin SA. Changing epidemiology and emerging risk groups for pertussis. Can Med Assoc J. 2006;174(4):451–452.
15. Vickers D, Ross AG, Mainar-Jaime RC, Neudorf C, Shah S. Whole-cell and acellular pertussis vaccination programs and rates of pertussis among infants and young children. Can Med Assoc J. 2006;175(10):1213–1217.
16. Bettinger JA, Halperin SA, De Serres G, Scheifele DW, Tam T. The effect of changing from whole-cell to acellular pertussis vaccine on the epidemiology of hospitalized children with pertussis in Canada. Pediatr Infect Dis J. 2007;26(1):31–5.
17. Greenberg DP, Doemland M, Bettinger JA, Scheifele DW, Halperin SA, Waters V, et al. Epidemiology of pertussis and haemophilus influenzae type b disease in Canada with exclusive use of a diphtheria-tetanus-acellular pertussis- inactivated poliovirus-haemophilus influenzae type b pediatric combination vaccine and an adolescent-adult

tetanus-diphtheria-acellular pertussis vaccine: Implications for disease prevention in the United States. Pediatr Infect Dis J. 2009;28(6):521–8.
18. Fisman DN, Tang P, Hauck T, Richardson S, Drews SJ, Low DE, et al. Pertussis resurgence in Toronto, Canada: a population-based study including test-incidence feedback modeling. BMC Public Health. 2011;11(1):694.
19. Chambers C, Skowronski D, Hoang L, Guiyun LH, Fritz C, Gustafson R, et al. Pertussis Surveillance Trends in British Columbia, Canada, over a 20-year Period: 1993-2013. Canada Commun Dis Rep. 2014;40(3):31–41.
20. New Brunswick Department of Health. Pertussis outbreak investigation report - April 2014 [Internet]. Available from: https://www2.gnb.ca/content/dam/gnb/Departments/h-s/pdf/en/CDC/HealthProfessionals/PertussisReport.pdf
21. Deeks S, Lim G, Walton R, Fediurek J, Lam F, Walker C, et al. Prolonged Pertussis Outbreak in Ontario Originating in an Under-immunized Religious Community. Canada Commun Dis Rep. 2014;40(3):42–9.
22. Liu XC, Bell CA, Simmonds KA, Svenson LW, Fathima S, Drews SJ, et al. Epidemiology of pertussis in Alberta, Canada 2004–2015. BMC Public Health. 2017;17(1):539.
23. Domenech de Cellès M, Magpantay FMG, King AA, Rohani P. The pertussis enigma: reconciling epidemiology, immunology and evolution. Proceedings Biol Sci. 2016;283(1822).
24. Foley AR. A three-year experiment with combined diphtheria toxoid-pertussis vaccine. Can J Public Heal / Rev Can Sante'e Publique. 1946;37(7):259–67.
25. Little GM. Pertussis immunization in Edmonton. Can J Public Health. 1946;37:329-331.
26. Galanis E, King A, Varughese P. The Changing Age and Seasonal Profile of Pertussis in British Columbia, Not Canada. J Infect Dis. 2002;186(10):1537–8.
27. Skowronski DM, De Serres G, Patrick DM, Halperin S. Reply. J Infect Dis. 2002;186(10):1538.
28. Halperin SA, De Serres G. Has the change to acellular pertussis vaccine improved or worsened pertussis control? Can Med Assoc J. 2006;175(10):1227 LP – 1227.
29. WHO WHO SAGE pertussis working group: Background paper. 2014;(April):82. Available from: http://www.who.int/immunization/sage/meetings/2014/april/1_Pertussis_background_FINAL4_web.pdf
